# Drought Tolerant Capability of Pineapple [*Ananas comosus* (L.) Merr] Plant Microbiome

**DOI:** 10.21315/tlsr2025.36.1.4

**Published:** 2025-03-30

**Authors:** Rahayu Fitriani Wangsa Putrie, I Nyoman Pugeg Aryantha, Iriawati, Sarjiya Antonius

**Affiliations:** 1 Research Center for Applied Microbiology, National Research and Innovation Agency. JL. Raya Bogor KM. 46 Cibinong 16911, West Java, Indonesia; 2 School of Life Sciences and Technology, Bandung Institute of Technology. JL Ganesa 10, Bandung 4013, West Java, Indonesia; 3 Institut Teknologi Sumatera, Jl. Terusan Ryacudu, Way Huwi, Kec. Jati Agung, Kab. Lampung Selatan, Lampung 35365, Indonesia

**Keywords:** *Bacillus*, Drought Tolerant, Endophyte, Pineapple, Rhizobacteria

## Abstract

The microbiomes of Indonesian pineapple plants cover drought-resistant microorganisms that have not yet been studied. Therefore, this research aims to analyse the pineapple’s endophytic and rhizobacteria capability to survive and support the plant in drought. The screening used *polyethylene glycol* (PEG) 6000 with specific osmotic pressures as a form of stress simulation. The isolates were further tested for their production of exopolysaccharides (EPS) and growth hormones (IAA), survival at high temperatures and salinity and other vital, drought-tolerant factors. Based on PEG 6000 analysis with certain osmotic pressure, about 13 isolates could survive at −0.73 MPa. Some isolates can produce EPS up to 89.23 mg/mL at −0.73 MPa, survive at 10% salinity, at a temperature of 50°C, pH 4 and produce IAA up to 7.5 ppm on medium. Most isolates can improve corn seedlings’ growth quality and produce ACC deaminase and catalase enzymes. Isolate BDO 8 and BAO 5 showed more constant results compared with others. Based on the 16S rRNA gene, these isolates were identified as *Bacillus cereus* strain ATCC 14579T.112 and *Bacillus cereus* strain WHX1 with 99.91% and 100% sequence similarities, respectively. These findings suggest that these isolates could be developed as bioinoculant candidates for use in dry agricultural areas.

**Highlights**

- The microbiomes of Indonesian pineapple plants covers drought resistant microorganism.
- The pineapple’s microbiome, especially endophytic and rhizobacteria capability to survive and support the plant in drought.
- Indigenous isolates can be developed as bioinoculant candidates for use in dry agricultural areas.

## INTRODUCTION

Plants are host to microbial communities that live below and above the ground. These microbes are obtained vertically from seeds and horizontally from the environment (Frank *et al*. 2017). The community of microorganisms that inhabit plants and play important roles is known as the plant microbiome. These microorganisms can be isolated and formulated into biological products for sustainable agriculture (Santos & Olivares 2021). Microbial symbion spread in all parts of the plant, both inside the tissue (endophytes), rooting areas (rhizobacteria) and the surface of parts (Chen *et al*. 2017). Endophytes have been known to spend most of their life cycle in plant tissue and have no detrimental effects on host plants (Kandel *et al*. 2017). Rhizobacteria are naturally present in plant roots and have various mechanisms to stimulate growth, therefore, known as growth-promoting rhizobacteria (PGPR) (Jeyanthi & Kanimozhi 2018).

The inoculation of plants with endophytes and rhizobacteria from dry land can increase the hosts’ fitness and growth (Asaf *et al*. 2017; Niu *et al*. 2018). These microbes can live symbiotically, increasing host plants’ tolerance to abiotic and biotic pressures (Kandel *et al*. 2017; Fahad *et al*. 2014). Endophytes and rhizobacteria can produce phytohormones and plant exopolysaccharides (EPS) to adapt at 9% NaCl, high osmotic pressure [20% polyethylene glycol (PEG)] and temperature at 48°C. These characteristics are critical factors in the mechanism of tolerance to drought (Asaf *et al*. 2017; Sandhya *et al*. 2009; Marasco *et al*. 2012).

Regarding drought, about 41% of earth’s terrestrial surface is dry land (Lacerda-Junior *et al*. 2019). Global warming and climate change make drought-affected areas more widespread and become significant challenges for future agriculture (Huang *et al*. 2015; Hone *et al*. 2021). In nature, harsh environmental conditions have shaped the type of living ecosystem, including plants. Some plant species are well adapted to arid and drought ecosystems. Pineapple is one type of plant that can survive in drought conditions. As a species of the *Bromeliaceae* family, it has a specific metabolic pathway that supports the ability to survive in a dry environment, namely crassulacean acid metabolism (CAM) (Davis *et al*. 2019). This plant can be a potential source of a microbial candidate tolerant to drought. Furthermore, pineapple bacteria are already accustomed to dry conditions. Microbes in host plant tissue that live in drought conditions are constantly exposed to low water availability (Nobel 2006).

The diversity data from the pineapple microbiome, specifically endophytic and rhizosphere originating from semi-arid regions in East Nusa Tenggara, Indonesia, has been reported previously. Culturable bacteria have been successfully isolated, obtaining 117 isolates from pineapple plants. A total of 88 were endophytic bacteria, while 29 were rhizobacteria (Putrie *et al*. 2020). These isolates can be developed as bioinoculants for agricultural areas exposed to drought conditions. It is important to note that there is no research to explore the ability of pineapple bacteria to improve plant survival. Therefore, it is necessary to evaluate the ability of these isolates to tolerate drought.

## MATERIALS AND METHODS

### Screening of Drought Tolerant Isolates

Screening was carried out to select isolates that are tolerant to drought. The initial step was to make Nutrient Broth (NB) (Oxoid™, UK) medium containing *polyethylene glycol* (PEG) 6000 at various levels of osmotic pressure. The concentration of PEG 6000 that was used on this test was consistent with the equations of Michel and Kauffman (1973), from 0 MPa, −0.5 MPa, −0.73 MPa, −1 MPa, −1.5 MPa up to −2 MPa. The inoculated medium was incubated at 30°C with a shaking of 130 rpm, and the growth was measured spectrophotometrically based on optical density at a wavelength of 570 nm. Isolates that were able to grow at −0.73 MPa were categorised as drought-tolerant (Sandhya *et al*. 2009).

### Isolates Selection Based on Exopolysaccharides Production

Exopolysaccharides (EPS) productions were tested quantitatively using media added with PEG 6000 at a certain concentration level. The incubated culture was centrifuged to separate the supernatant and pellet. The supernatant was mixed with 3 mL of absolute cold alcohol and precipitated at 4°C overnight. Subsequently, the solution was centrifuged for 15 min at 10,000 rpm. The precipitate obtained measured the quantity of EPS through the Dubois method.

### Screening of Isolates Based on Survival at High Temperature

Bacterial isolates were tested for their resistance at various temperatures, namely 30°C, 40°C, 50°C and 60°C, using NB medium. Afterward, the inoculated medium was incubated for 5 days at 30°C, and the test was conducted with three replicates. The growth of isolates was observed by measuring optical density at wavelengths ƛ 570 nm.

### Screening of Isolates Based on High Salinity Stress

Endophytic and rhizosphere bacteria were tested for their resistance under high salinity conditions at various concentrations, namely 5%, 10% and 15%. This concentration was added to the NB medium by adding sodium chloride (NaCl), and incubation was carried out for 5 days at 30°C. Testing was performed with 3 replicates, and the growth of isolates was observed by measuring optical density at wavelengths ƛ 570 nm.

### Screening of Isolates Based on Low pH Stress

The isolates were tested for resistance under acidic conditions (low pH) at various concentrations by adding hydrochloric acid (HCl) to the medium. The acidity levels tested were at pH 2, 3, 4 and 5 with three replicates. The isolates that were able to grow at the high acidity level were categorised as tolerant to acidic conditions.

### Screening of Isolates Based on Indole Acetic Acid (IAA) Production

Test isolates were grown on an NB medium that was added with 1 mM tryptophan. Furthermore, about 1 mL of the suspension culture was centrifuged at 10,000 g for 20 min. The supernatants obtained were transferred to a test tube and a total of 4 mL of Salkowsky reagent (150 mL concentrated H_2_SO_4_, 250 mL distilled water and 7.5 mL 0.5 M FeCl_3_) were added. The mix solution was incubated for 1 h at 30°C in dark conditions. Meanwhile, absorbance measurements were carried out at a wavelength of 520 nm. The absorbance value was converted to IAA concentration based on the equation obtained from the standard curve.

### In vitro Growth Stimulation Test

Drought-tolerant isolates were tested for their ability to stimulate plant growth in vitro on maize seedlings. The sprouts were selected as a bio-assay model because maize has a more uniform growth ability, is relatively fast, and is widely used as a test plant (Putrie *et al*. 2013; Asova *et al*. 2018). The initial stage was surface sterilisation by using Somasegaran and Hoben (1994) method. The seeds were soaked in distilled water for 24 h to speed up germination and placed in a Petri dish lined with filter paper. The next stage was to transfer the seeds to Petri dishes containing 1% water agar media. Each germinated seed was inoculated with 100 μL of isolate suspension with a total cell density of 10^9^ cells/mL. Seedlings not given bacterial suspension were used as controls, and incubation was carried out for 7 days at 28°C and dark conditions (Dey *et al*. 2004). Growth parameters observed were stem length, root length, number of lateral roots and number of leaves. One-way analysis of variance (ANOVA) was used with the Tukey test at a 95% confidence level by IBM SPSS Statistic version 25 software (IBM Corp, USA).

### Matriculation for Potential Screening Isolates

A total of three isolates were selected from each seven parameters as the critical factors of drought tolerance, and then a table was made. The parameter table was a reference for creating a ranking test result matrix table. Potential isolates were selected based on the highest value obtained in the reference table.

### Analysis of 1-aminocyclopropane-1-carboxylic acid (ACC) Deaminase Enzyme Production

The five best isolates judged to be in the drought-tolerant criteria were based on the ranking matrix and tested for their ability to produce the ACC deaminase enzyme. This test was conducted by inoculating isolates in Dworkin-Foster (DF) minimal salt medium with the addition of 1-aminocyclopropane-1-carboxylate (*ACC*) substrate and DF media with ammonium sulfate (Am S) as ACC substrate replica. The ability to produce the ACC deaminase enzyme was observed from the growth of the isolate colonies on the media. The control was DF media without adding *ACC* or Am S substrate (Ali *et al*. 2014).

### Analysis of Catalase Enzyme Production

The five isolates used in the subsequent ACC deaminase enzyme production test were also tested for their ability to produce catalase enzyme. This enzyme functions as a cellular detoxifier or avoids oxidative damage caused by hydrogen peroxide (H_2_O_2_). This test is conducted by mixing a loop of bacterial colonies with catalase reagent on a slide, and the suspension is stirred continuously with a sterile toothpick. Results categorised as positive can produce catalase enzyme when air bubbles are formed (Reiner 2010).

### Identification Based on 16S Ribosomal RNA (rRNA) Gene

The two bacterial isolates which showed the best drought-tolerant ability were identified based on 16S rRNA gene sequences. Genomic DNA was extracted using the Quick-DNA™ Fungal/Bacterial Miniprep Kit (Zymo Research, USA, catalog no. D6005). Furthermore, 16S rRNA genes were amplified using the MyTaq HS Red Mix kit (Bioline, USA, BIO-25047). Universal primers used in the PCR process are 27 F (5′ AGAGTTGGCCTGGCTCAG-3′) and 1492 R (5′-GGTTACCTTGTTACGACTT-3′). The purification of the PCR product used was Zymoclean™ Gel DNA Recovery Kit (Zymo Research, USA, catalog no. D4001). In addition, sequencing was performed bi-directional sequencing by Novogen™ through Genetics Science Indonesia. DNA sequences were analysed by comparing to the GenBank database using the BlastN programme (http://www.ncbi.nlm.nch.gov) from the National Center for Biotechnology Information. A phylogenetic tree was created using Mega X software with Construct Test Neighbour-Joining Tree.

## RESULTS

### Drought Tolerant Isolates

**Figure 1:**
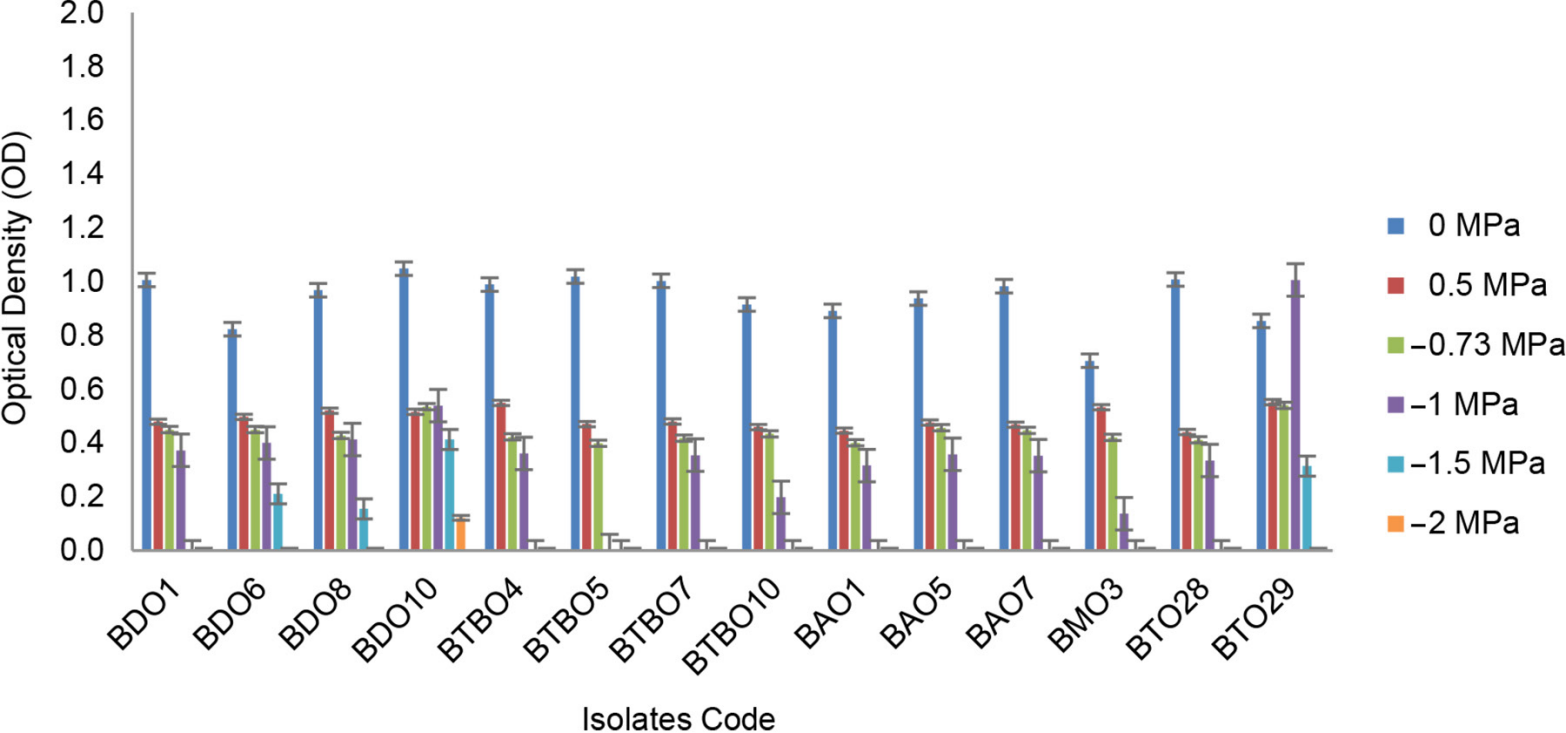
Capability of isolates growth on mediums with different osmotic levels (error bars ± SD).

Based on the test results, 13 of 117 isolates derived from leaves (4), fruit stalks (3), roots (3), bacteria (1) and the rhizosphere (2) were drought-tolerant. These screening results are shown in Fig. 1. BDO 10 is an isolate with the best drought tolerance ability that can still grow (OD > 0.4) on a medium with an osmotic pressure of −1.5 MPa.

### Isolate Capability for Exopolysaccharides (EPS) Production

**Figure 2:**
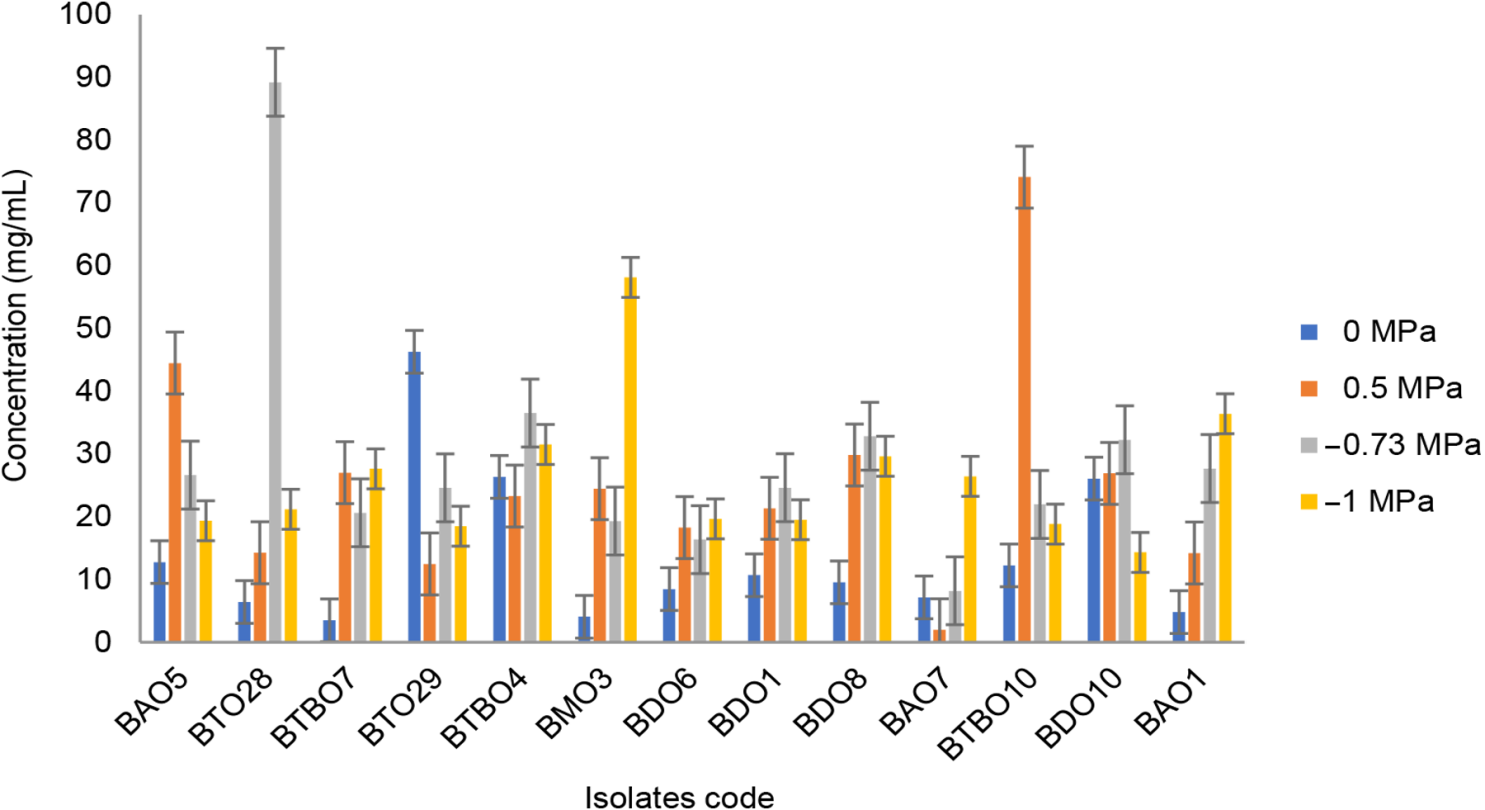
Exoplysaccharide production of drought tolerant isolates on mediums with certain osmotic levels (error bars ± SD).

The isolates categorised as drought-tolerant were tested for their ability to EPS production. The ability to produce EPS by isolates is shown in Fig. 2. The test isolates were able to produce EPS under various conditions depending on the level of osmotic stress. Isolate BTO 28 produced the highest EPS production at an osmotic pressure of −0.73 MPa at 89.227 mg/mL.

### Isolate Survival at High Temperatures and Salinity Pressures

**Figure 3:**
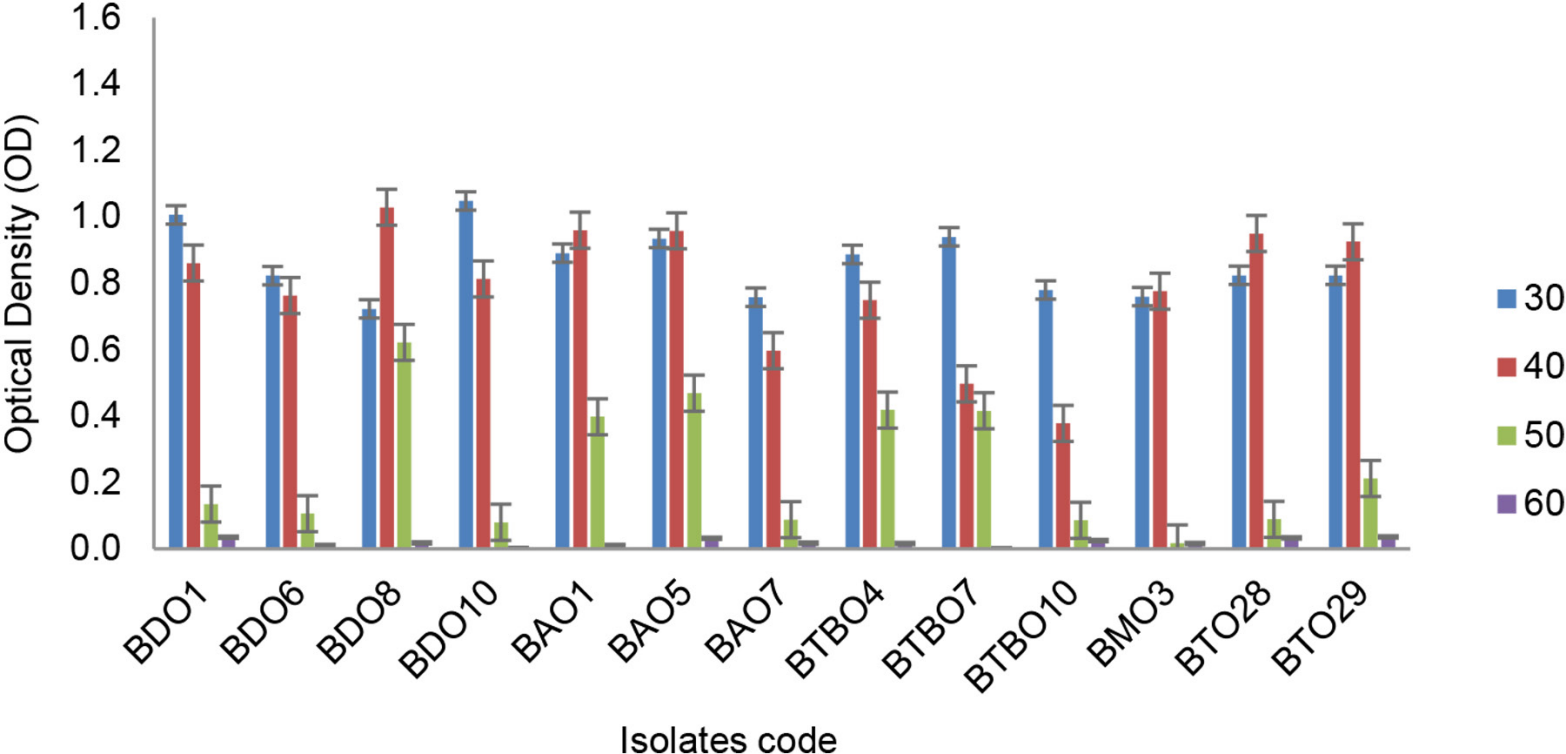
Capability of drought tolerance isolates growth on mediums with certain temperature levels (error bars ± SD).

**Figure 4:**
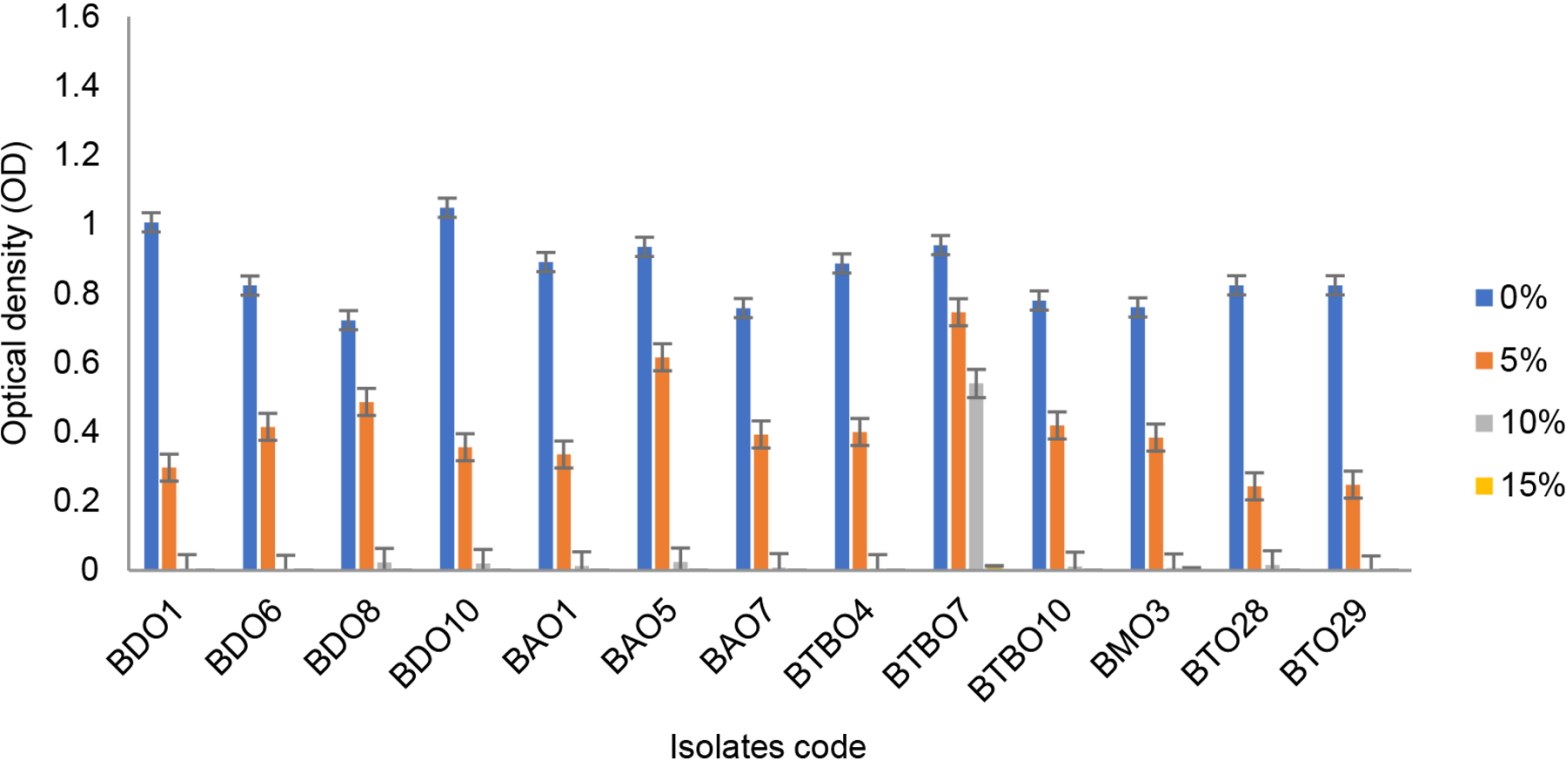
Capability of drought tolerance isolates growth on mediums with certain salinity levels (error bars ± SD).

The ability of isolates to grow on media with specific temperature and salinity pressures is shown in Figs. 3 and 4, respectively. Most of these isolates were still able to grow well at 40°C. BDO 8 can also survive up to a temperature of 50°C with an OD value above 0.4. On the other hand, BTBO 7 is an isolate with the best salinity tolerance ability that can still grow (OD > 0.4) on medium with 10% salinity pressure.

### Isolate Survival at Low pH Pressures

**Figure 5:**
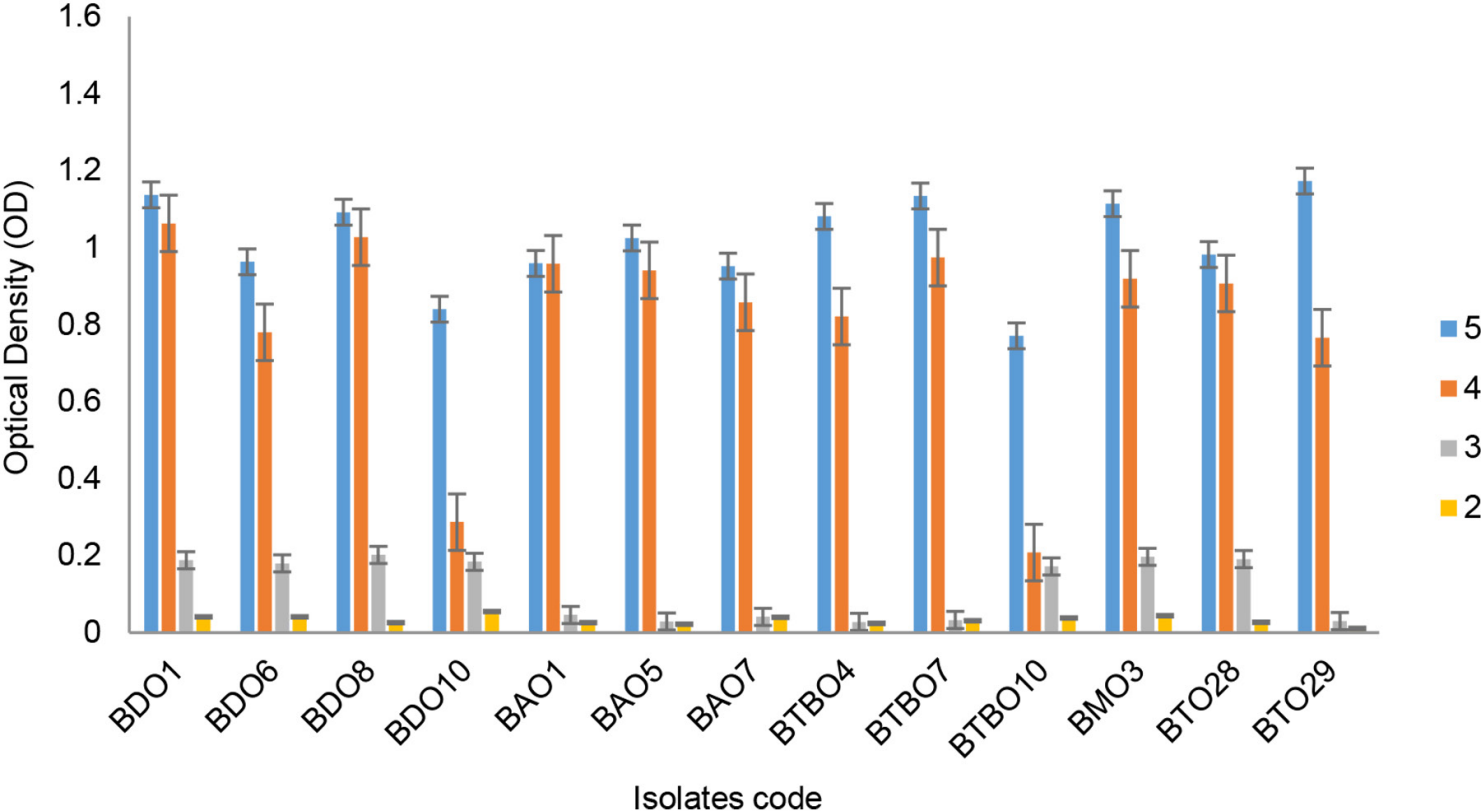
Capability of drought tolerance isolates growth on mediums with certain pH levels (error bars ± SD).

The ability to grow on media with specific pH pressures by isolates is shown in Fig. 5. Most of the isolates were still able to grow well at pH 4, and BDO 1 had the lowest pH tolerance ability. The isolates no longer grow appropriately at a pH of more than 4 (OD > 0.4).

### Isolate Capability to Produce Indole Acetic Acid

**Figure 6:**
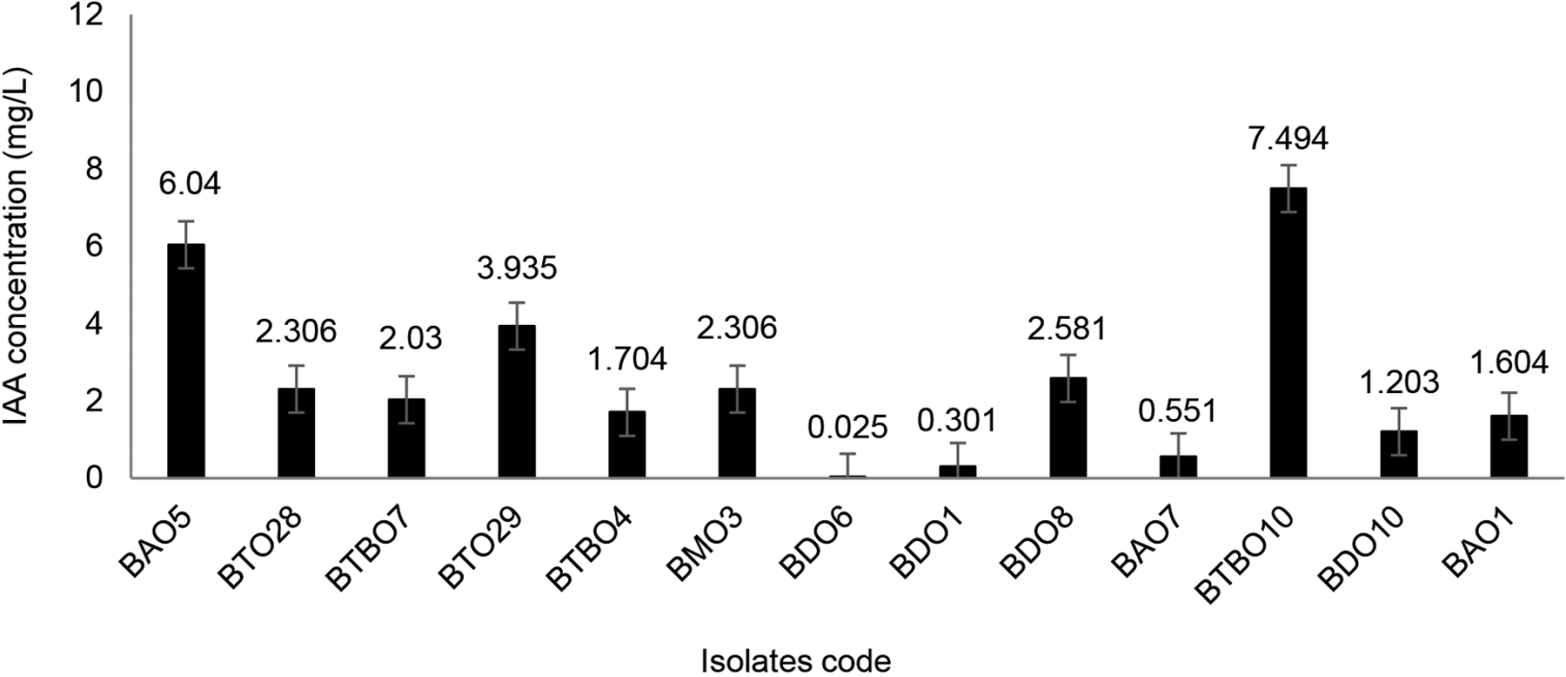
Capability of drought tolerance isolates to produce growth hormone of indole acetic acid (IAA) (error bars ± SD).

Fig. 6 shows the ability to produce the growth hormone indole acetic acid. The IAA hormone produced was in the range of 0.025 ppm–7.494 ppm, and the BTBO 10 isolate produced the highest amount.

### The Effect of Isolate on Maize Sprout Growth

The results obtained are shown in Table 1. Most inoculants increased the growth of corn sprouts on the five growth parameters compared to the control. BAO 7 is the best isolate from the in vitro test results.

### The Rank of Potential Isolates

The critical factors of drought tolerance isolates with the best ability were selected from seven test parameters. Table 2 shows the three best isolates. Five isolates with the highest scores were selected based on the scoring process.

### Analysis of ACC Deaminase and Catalase Enzyme Production

The five best isolates considered in the drought-tolerant criteria were based on the ranking matrix. Furthermore, they were tested for their ability to produce the ACC deaminase (Table 3) and catalase enzyme (Table 4). The test results in producing the ACC deaminase enzyme were shown by the ability to grow on the test medium. The isolates were able to grow on the three test media, both on Dworkin-Foster (DF) minimal salt medium with the addition of *ACC* substrate, DF medium with ammonium sulphate (Am S) as a replica of the *ACC* substrate and on the control, namely DF media without the addition of *ACC* and Am S substrates. The isolates also produced catalase enzyme following the formation of air bubbles after stirring with the reagents.

### Identification Based on 16S rRNA Gene

**Figure 7:**
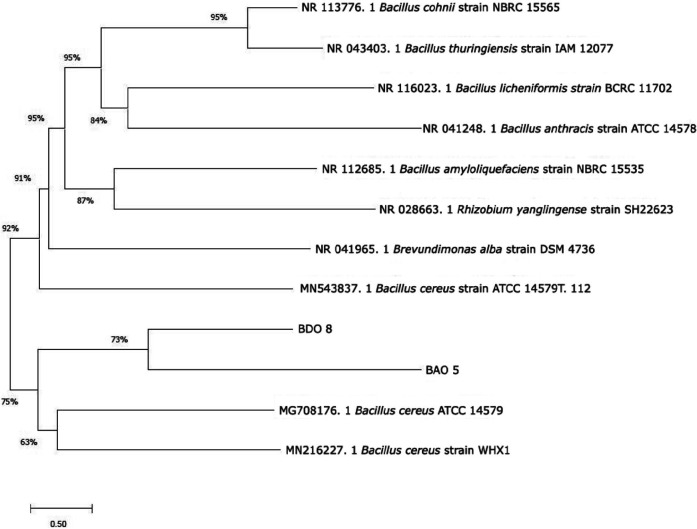
Phylogenetic tree based on 16S rRNA gene sequences from BDO 8 and BAO 5. The scale shows the evolutionary distance in branch length, while the number on the branch indicates the data coverage.

BDO 8 and BAO 5 were the best drought-tolerant isolates from the in vitro test screening results were identified as *Bacillus cereus* strain ATCC 14579T.112 and *B. cereus* strain WHX1, and the phylogenetic tree is shown in Fig. 7.

## DISCUSSION

PEG 6000 added to the media can bind water molecules and reduce the potential value as a simulation of drought stress (Putrie *et al*. 2013). BDO 10 is a potential isolate that can grow at osmotic pressure of −1.5 MPa obtained from the leaves of the pineapple plant. The leaves with slightly fleshy and waxy leaf morphology also affect the endophytic bacteria in their tissues. These bacteria are naturally exposed to low water content (Nobel 2006; Davis *et al*. 2019). Meanwhile, LTYR-11ZT was derived from the leaves of *Alhagi sparsifolia* Shap. in northwestern China (Chen *et al*. 2017).

Exopolysaccharides (EPS) is a structural component of the extracellular matrix in biofilms synthesised by cells in response to physiological, biotic and abiotic stresses (Marvasi *et al*. 2010). It was used as an indicator of the selection of drought-tolerant bacteria (Sandhya *et al*. 2009; Donot *et al*. 2011). Furthermore, it protects cells from drought, and heavy metals respond to host immune and produce biofilms to increase cell resistance in ecological niches (Ozturk & Aslim 2010). The bacterial tolerance to drought is directly proportional to the EPS produced (Putrie *et al*. 2013). The *Bacillus* group is the best EPS producer, specifically under stress. EPS (0.66 mg.mL–0.91 mg/mL) can be produced under normal and heat conditions (Mukhtar *et al*. 2020). Our finding on isolate BTO 28 with the highest EPS production at an osmotic pressure of −0.73 MPa was able to produce 89.227 mg/mL. With this capacity of EPS synthesis, isolate BTO 28 was not able to give the best effect on plant performance, meaning other factors are also important for plant growth.

High-temperature tolerance determines the selection of bioinoculant candidates for agricultural areas with dry conditions. It is crucial because the majority of temperatures on dry land are higher. Inoculation using thermotolerant isolates has been shown to reduce membrane damage and the activity of several antioxidant enzymes such as SOD, APX and CAT. In addition, it can increase the production of cellular metabolites such as proline, chlorophyll, sugars, starch, amino acids and proteins (Ali *et al*. 2011).

BDO 8, which is the best isolate from this test, is a Gram-positive bacteria that can survive in arid conditions. This is because the cell wall layer in their biofilm is thicker than Gram-negative, such as *B. subtilis* and *Pseudomonas aeruginosa*, which have cell walls of 55.4 nm and 2.4 nm, respectively. Moreover, Gram classification can correlate with cell envelope differences in bacteria. These differences give cells different properties, particularly their response to external stresses, including heat, UV radiation and antibiotics (Mai-Prochnow *et al*. 2016).

Drought soils have high salinity due to reduced leaching fraction. Plants growing on this soil experience osmotic stress and homeostatic disturbances due to salt accumulation (Machnado & Serralheiro 2017). The tolerance ability to high salinity is also related to the production of the resulting EPS. In the previous results, BTBO 7 was one of the isolates producing EPS. The resulting EPS can bind to cations, including Na^+^, decreasing salinity pressure (Ashraf *et al*. 2004). Bio-inoculation of EPS-producing *B. cereus* has significantly reduced electrical conductivity (EC), Na and Cl content by 35% (Hassan *et al*. 2018).

Besides high salinity conditions, inoculants for dry land were also exposed to low pH conditions associated with hydrogen ion concentrations in the soil. The level of acidity and alkalinity is progressive and requires microbial technology that provides increased tolerance to pH stress (Msimbira & Smith 2020). The dry tolerant isolates are included as candidate criteria because it was able to survive up to pH 4. The range of low pH criteria for acid-tolerant bacteria originating from the soil is 3.8–5.5 (Goswami *et al*. 2017).

Bacterial isolates from different host plants were used during the bioassay test (cross-inoculation). The use of bacterial isolates from different host plants may not be relevant, mainly when applied in the field. However, the results showed that they could still improve the agronomic growth parameters in the model plants. Putrie *et al*. (2013) also showed similar results, where a consortium of *Bacillus* and *Pseudomonas* formulas derived from the rhizosphere of soybean plants could increase the growth of maize plants. In addition, endophytic bacteria 4EA10.1 isolated from *Curcuma xanthorrhiza* is also known to increase root length and the number of leaves on rice seedling growth (Saryanah *et al*. 2021).

The IAA hormone plays a vital role in all aspects of plant growth and development, specifically in conditions of abiotic pressure. The IAA controls the plants’ signalling under these conditions. It can also increase nutrient uptake in limited nutritional conditions by modifying the root architecture appropriate for plants (Hassan *et al*. 2018). Root length determines the ability to absorb nutrients in the soil with a broad reach (Asova *et al*. 2018). This vital hormone makes IAA a marker indicator of drought-tolerant bioinoculant candidates.

The highest IAA produced by BTBO 10 isolate was 7.494 ppm. This value was still lower compared to endophytic isolates LK11, MPB5.3 and TP1, which were 12.31 ± 0.45, 6.8 ± 0.59, and 10.5 ± 1.02 M/mL in the culture broths (Asaf *et al*. 2017). However, for maize plants, these levels have increased growth. *Bacillus* and *Pseudomonas* inoculants with IAA production of 2.82–22.79 ppm were proven to increase the growth of maize seedlings in vitro tests. High IAA values can cause an inhibitory effect on plant growth (Putrie *et al*. 2013).

The enzymes 1-aminocylopropane-1-carboxylate (ACC) deaminase and catalase are essential in the drought tolerance mechanism. Expression of ACC deaminase is one of the characteristics of bacteria that can be associated with plants. This bacteria can reduce ACC and ethylene concentrations in plants and overcome damaging effects in various aspects of plant-microbial interactions, plant growth and development under stress conditions (Glick & Nascimento 2021). Catalase enzyme is also essential in the mechanism of increasing drought tolerance. These antioxidants are involved in endophytic-plant interactions to increase tolerance to hot and dry conditions (Xu *et al*. 2017). Catalase functions as a cellular detoxifier or avoids oxidative damage caused by hydrogen peroxide (H_2_O_2_) (Reiner 2010).

Based on the 16S rRNA gene, two bacterial isolates which showed the best drought-tolerant ability were identified. Identification is needed to ascertain the species obtained, and the gene is a conserved sequence area in all bacteria at different stages of evolution. The length is around 1,500 bp with nine hypervariable regions, namely V1–V9 (Winand *et al*. 2020). The primary bioinformatics tool for looking through similar gene sequence comparisons and genome search databases is Basic Local Alignment Search Tool (BLAST). The ranking of BLAST results depends on the length and quality of gene sequence treatment (Newell *et al*. 2013).

The BLAST result showed good scores for BDO 8 and BAO 5 isolates at 99.91% (Accession Number MN543837.1) and 100% (Accession Number MN216227.1), respectively. Using a phylogenetic tree approach, the isolates were in 1 clade with a coverage value of only 73%. This may be due to the NCBI database gene sequence used for *B. cereus* strain ATCC 14579T.112 and WHX1. Multiple sequences and fast alignments (FASTA) were used in making the phylogenetic tree. However, the results of BLAST analysis will be faster and more accurate than FASTA (Donkor *et al*. 2014).

*Bacillus* is the genus identified as the pineapple microbiome (Putrie *et al*. 2020). *Bacillus* sp. endophyte. Acb9 has been isolated from the *Ananas comosus* plant (Jayakumar *et al*. 2020) and pineapple peels (Raguati & Endri 2018). This research also obtained the same results, that the microbiome species identified from the *Ananas* plant were from the genus *Bacillus*, specifically *B. cereus*.

Several reports indicated that *B. cereus* is a pathogen (Messelhauber & Ehling-Schulz 2018; Gharib *et al*. 2020; Rodrigo *et al*. 2021), but not all isolates are pathogenic. Arif *et al*. (2017) showed that a particular species has the potential as a plant growth promoter. *B. cereus* GS6 is reported to increase the efficiency of soybean symbiosis and has great potential in dissolving and mobilising phosphate. Inoculants of *B. cereus* IB311 were also reported to prevent disease and increase production in *Arachis hypogaea* var. Koushal, G201 and *Sesamum indicum* var. Kanak (Banerjee *et al*. 2018). Furthermore, *B. cereus* was a biological control agent against bacterial heart rot in pineapple (Husin & Sapak 2022). The results of previous experiments (Table 1 and Fig. 7) also showed that the isolates were not pathogenic because they did not cause disease symptoms in the bioassay test.

*B. cereus* is the best dry-tolerant isolate and can form spores under unfavourable environmental conditions (Li *et al*. 2017). Furthermore, it is vital in mitigating negative effects on plant growth and development. The isolate can increase the physiological and biochemical endurance of tomato varieties that grow under heat stress with exopolysaccaride production (Mukhtar *et al*. 2020). Most endophytic bacteria isolated are from the genus *Bacillus*, known to have the ability to regulate gene expression responsive to drought and modulate DNA methylation processes. It significantly affects plant metabolism, such as increasing sucrose, asparagine fructants, glutamic acid and glutamine (Gagne-Bourque *et al*. 2016).

## CONCLUSION

The test results proved that endophytic and rhizobacteria plant microbiomes culturable from pineapple plants have drought-tolerant properties. BDO 8 and BAO 5 have the characteristics of the best drought-tolerant isolates. Therefore, these isolates have strong potential to be developed as bio-inoculant candidates for drought-affected land.

## Figures and Tables

**Table 1:** Effect of inoculant on maize seedling growth.

Isolates code	RL	SL	LL	NRL	NL
Control	7.54 ± 0.55	3.10 ± 0.82	4.70 ± 0.57	5.40 ± 1.34	1.00 ± 0.00
BAO 1	13.90 ± 1.14^a^	6.80 ± 1.09^a^	6.00 ± 0.35	9.00 ± 2.00^a^	1.40 ± 0.55
BAO 5	20.70 ± 3.67^a^	5.80 ± 1.79^a^	6.40 ± 0.82	5.60 ± 1.34	1.80 ± 0.45
BAO 7	21.60 ± 2.51^a^	6.46 ± 0.69^a^	7.40 ± 2.38^a^	8.00 ± 1.58	2.00 ± 0.71
BDO 1	19.00 ± 1.90^a^	5.10 ± 1.39	6.80 ± 2.92	8.40 ± 0.55	1.80 ± 0.45
BDO 6	16.90 ± 0.42^a^	6.60 ± 0.42^a^	6.10 ± 1.67	7.80 ± 1.09	1.80 ± 0.45
BDO 8	11.90 ± 3.09	7.00 ± 1.00^a^	4.90 ± 0.42	7.60 ± 1.52	1.60 ± 0.55
BDO 10	10.94 ± 3.27	5.50 ± 0.79^a^	6.26 ± 2.35	8.40 ± 0.95	1.80 ± 0.45
BMO 3	16.10 ± 2.36^a^	5.50 ± 0.50^a^	6.90 ± 1.14	7.80 ± 1.64	2.00 ± 0.00
BTO 28	10.10 ± 3.81	5.26 ± 0.84^a^	5.30 ± 0.67	6.80 ± 1.09	1.40 ± 0.89
BTO 29	15.20 ± 1.04^a^	5.76 ± 0.43^a^	5.20 ± 0.67	8.20 ± 1.64	2.00 ± 0.00
BTBO 4	10.80 ± 1.95	4.76 ± 0.91	5.54 ± 1.36	7.00 ± 1.22	1.20 ± 0.45
BTBO 7	11.20 ± 3.13	6.20 ± 0.84^a^	4.40 ± 0.82	7.40 ± 1.67	1.20 ± 0.45
BTBO 10	15.10 ± 5.27^a^	6.50 ± 0.50^a^	6.76 ± 1.66	7.40 ± 0.55	1.80 ± 0.45
Rata2 ± SD	14.36 ± 4.79	5.74 ± 1.30	5.90 ± 1.61	7.49 ± 1.63	1.63 ± 0.65

*Notes*:

* mean values with superscript letters within each column denote significant (*p* < 0.05). differences with control.

RL = Root Length (cm); SL = Sheath Length (cm); LL = Leaf Length (cm); NRL = Number of Lateral Roots; NL = Number of Leaves

**Table 2:** The best three isolates of each test category.

Test category						

Drought tolerance	EPS production	High salinity	High temperature	Low pH	IAA production	Growth promoter
BDO 10	BTO 28	BTBO 7	BDO 8	BDO 1	BTBO 10	BAO 7
BTO 29	BTBO 4	BAO 5	BAO 5	BDO 8	BAO 5	BAO 5
BDO 8	BDO 8	BDO 8	BTBO 4	BTBO 7	BTO 29	BDO 6

**Table 3:** Capability of isolates for their ability to produce the 1-aminocyclopropane-1-carboxylic acid (ACC) deaminase enzyme.

Isolates codes	Growth capability								

	DF + ACC			DF			DF + ammoniumsulphate		
		
	Not grown	Grown	Well grown	Not grown	Grown	Well grown	Not grown	Grown	Well grown
BDO 8			++		+			+	
BAO 5			++			++			++
BTO 29		+				++			++
BTBO 4			++		+				++
BTBO 7			++		+				++
BTBO 7			++		+				++

*Note*: DF = Dworkin Foster. The ability of isolates to produce ACC deaminase enzymes was categorised based on the growth of isolates on various medium: Dworkin Foster (DF) + *ACC* substrate, medium Dworkin Foster (DF), medium Dworkin Foster (DF) + ammonium sulfate (Am S).

**Table 4:** Capability of isolates for their ability to produce catalase enzyme.

Isolates codes	Catalase			

	Reaction category			

	Not reactive	Reactive	More reactive	Most reactive
BDO 8			++	
BAO 5				+++
BTO 29		+		
BTBO 4			++	
BTBO 7			++	

*Note*: The ability of isolates to produce catalase enzymes was categorised based on reaction time and the formation of air bubbles after stirring with reagents.
